# Application and validation of AI-assisted 3D-Printed gastroduodenal anatomical variation models in specialized nursing training

**DOI:** 10.3389/fbioe.2026.1769764

**Published:** 2026-06-19

**Authors:** Hao Li, Shaohua Sun, Xiang Mao, Wenbo Zhou

**Affiliations:** Department of Hepatobiliary and Pancreatic Surgery, Sinopharm Dongfeng General Hospital Affiliated to Hubei University of Medicine, Shiyan, Hubei, China

**Keywords:** 3D printing, AI-assisted modeling, anatomical variation, biliary system, ERCP, specialized nurses

## Abstract

**Objective:**

To investigate the application effectiveness of AI-assisted 3D printing technology for modeling anatomically abnormal upper gastrointestinal-biliary systems in ERCP specialized nurse training.

**Methods:**

A total of 179 nursing staff were randomly assigned to an experimental group (n = 84) receiving AI-assisted 3D-printed biliary models or a control group (n = 95) receiving traditional instruction. Post-training outcomes included theoretical and practical examinations, a five-point Likert questionnaire (covering teaching model, content, learning interest, operational skills, and problem-solving abilities), and expert evaluations. Statistical analyses compared the two groups.

**Results:**

The experimental group showed significantly higher theoretical and practical assessment scores than the control group (both *p* < 0.05). In the teaching effectiveness feedback, the experimental group scored significantly higher in teaching model, stimulating learning interest and enhancing operational skills (all *p* < 0.05). ERCP physician feedback also revealed significantly higher satisfaction in cooperation awareness, procedural accuracy, and teamwork awareness for the experimental group (all *p* < 0.05).

**Conclusion:**

AI-assisted 3D printing technology demonstrated preliminary advantages in ERCP nurse training, but its broader application requires validation in larger, multi-center trials with long-term outcomes.

## 1 Introduction

With the rise of endoscopic technology, digestive surgery has experienced vigorous development. The clinical value of endoscopic retrograde cholangiopancreatography (ERCP) in diagnosing and treating biliopancreatic diseases has been widely recognized (Shen et al., 2024). Initially focused primarily on observation and diagnosis, ERCP has evolved with technological advances into a comprehensive endoscopic diagnostic and therapeutic technique, now representing the most important modality for managing biliopancreatic diseases (Mill et al., 2022). Its prominent “minimally invasive” advantages have continuously expanded its application scope and indications, with widespread use in bile duct stones, strictures, tumors, and cases of undiagnosed obstructive jaundice and unexplained pancreatic duct dilation. Its unique value in treating surgical obstructive diseases is particularly notable (Li et al., 2025).

However, ERCP procedural success is highly dependent on individual anatomical conditions. Studies show that anatomical abnormalities are key factors leading to difficult ERCP cannulation, failure, and complications. Common abnormalities include gastroduodenal angle variations, duodenal papilla position variations (such as intradiverticular or deep fold locations), and severe deformities of the duodenal bulb or descending portion (such as post-surgical strictures or external compression-induced distortion) (Oh et al., 2024). These variations directly prevent stable endoscope positioning against the wall and obscure papilla visualization, and misalign cannulation instrument axes with bile duct trajectories, significantly increasing papilla cannulation failure rates. To overcome difficulties, operators often must repeatedly attempt using pre-bent catheters, specialized cutting knives, or auxiliary guidewires, which not only substantially prolongs operative time and radiation exposure but also readily causes papillary edema, injury, or even perforation (Chen Q. et al., 2025). Therefore, ERCP places extremely high demands on the clinical competence of endoscopy nurses. Insufficient clinical experience and inadequate specialized training among endoscopy nurses are also major causes of prolonged operative times and increased complications (Chen D. et al., 2025).

Currently, specialized digestive endoscopy nurse training abroad is relatively well-established, but domestically it remains in a developmental stage without a systematic, large-scale training framework. As ERCP endoscopic minimally invasive technology continues to develop, requirements for professionalism, refinement, and meticulousness among endoscopy center nurses constantly increase, making the cultivation of professional endoscopy nurses who can adapt to technological development needs an urgent practical issue. How to rapidly train junior endoscopy nurses into nursing professionals capable of expertly managing common difficult ERCP cannulations represents an important challenge for endoscopy nursing managers.

In this context, 3D printing technology provides a new pathway for simulation training. 3D-printed models can realistically simulate patients' duodenal structural states and create duodenal and biliary anatomical abnormality models at specific sites for repeated trainee practice, creating conditions for improving biliary cannulation success rates. 3D printing provides artificial biliary system models allowing continuous observation and repeated operation, creating realistic operative environments and excellent operational feedback for trainees. For patients with anatomical abnormalities, the application value of 3D-printed models becomes even more prominent: they can high-fidelity reproduce individualized complex anatomical variations, such as gastroduodenal angle variations (excessive acute angulation), peripapillary diverticula, or abnormal bile duct branching, providing trainees with precise rehearsal for “difficult ERCP cannulation” that standardized simulators cannot achieve, thereby systematically enhancing endoscopy nurses' cognitive preparation and operational adaptability for real clinical challenges.

Therefore, this study will establish personalized 3D models of the upper gastrointestinal-biliary system based on CT imaging data from patients with real anatomical abnormalities, utilizing AI-assisted technology, and apply them to continuing education training for ERCP endoscopy nurses. The aim is to improve nurses' clinical competence through this innovative training model and further enhance endoscopic nursing care quality.

## 2 Clinical data and methods

### 2.1 General characteristics

Nursing personnel (total 179) working or rotating for training at the ERCP Endoscopy Center of Sinopharm Dongfeng General Hospital (referred to as “this center”) from January 2020 to January 2025 were selected as study subjects. Inclusion criteria: studying at this center and participating in endoscopic nursing work for more than 3 months; capable of independently assisting with endoscopic minimally invasive surgery; college degree or above; able to independently complete knowledge mastery assessment forms. This training obtained informed consent from all nurses and was approved by the hospital ethics committee. Exclusion criteria: age >45 years; not participating in endoscopic minimally invasive surgery assistance; unable to independently assist with endoscopic minimally invasive surgery; part-time endoscopy nurses. Subjects were divided into an Experimental Group (E.G.,) and Control Group (CG) using stratified block randomization. (stratification factor: prior ERCP assisting experience, <50 vs. ≥50 cases; block size = 4). The randomization sequence was generated by an independent statistician using R software (seed = 20240101) and concealed in sequentially numbered, opaque, sealed envelopes prepared by a non-study assistant. Group assignment was revealed by opening the next envelope after enrollment. Blinding: Due to the nature of the intervention, participants and instructors could not be blinded. However, all outcome assessors (physicians evaluating performance, examiners grading theoretical/practical tests) and the data analyst were kept blinded to group allocation using unique study codes. Experimental group: 84 participants, including five male and 79 female nurses; age (29.1 ± 6.4); education level: 44 college degree, 40 bachelor’s degree; work experience: 25 with less than 5 years, 44 between 5 and 10 years, 16 over 10 years. Control group: 95 total, including seven male and 88 female nurses; age (29.5 ± 6.1); education level: 49 college degree, 46 bachelor’s degree; work experience: 27 within 5 years, 49 between 5 and 10 years, 19 over 10 years. General data comparison between the two groups showed no statistically significant differences (*p* > 0.05), indicating comparability. Additionally, pre-enrollment theoretical and practical assessments of all 179 subjects showed theoretical assessment scores (65.39 ± 2.76 vs. 64.53 ± 3.28, t = 1.92, *p* > 0.05) and practical assessment scores (72.68 ± 5.61 vs. 73.73 ± 6.59, t = −1.149, *p* > 0.05) with no statistically significant differences (see Table 1).Participant enrollment was terminated at a predefined calendar date (31 January 2025) rather than after completing all blocks. Therefore, the last block (block size = 4) was not fully recruited, resulting in slightly unequal group sizes (84 vs. 95). Randomization and allocation concealment were maintained throughout, and baseline characteristics remained balanced between groups.The study protocol was approved by the Ethics Committee of Sinopharm Dongfeng General Hospital (Approval No. LW-2025–04).

**TABLE 1:** Comparison of pre-training theoretical and practical assessment scores between groups.

Statistic	Pre-training theoretical score	Pre-training practical score
EG (n = 84)	65.39 ± 2.76	72.68 ± 5.61
CG (n = 95)	64.53 ± 3.28	73.73 ± 6.59
MD	0.87	−1.05
95% CI of MD	(-0.02, 1.76)	(-2.85, 0.75)
Cohen’s d	0.288	−0.172
95% CI of d	(-0.008, 0.583)	(-0.466, 0.122)
t	1.92	−1.149
df	176.6	176.7
P-value	0.0565	0.2521

EG: experimental group; CG: control group; MD: mean difference; CI: confidence interval. Comparison between groups, *p* < 0.05.

### 2.2 Research methods

**FIGURE 1:**
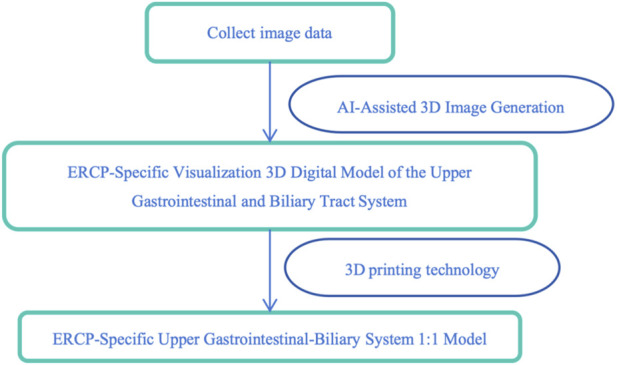
Flowchart of AI-Assisted ERCP-Specific upper gastrointestinal-biliary system 3D printing model.

**FIGURE 2:**
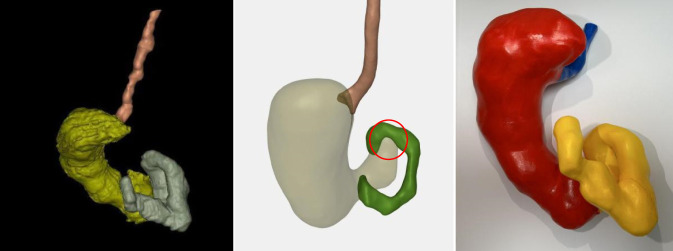
Upper gastrointestinal-biliary system digital modeling images, AI-Denoised images, and 3D-Printed anatomical abnormality physical model (excessive acute gastroduodenal angle).

**FIGURE 3:**
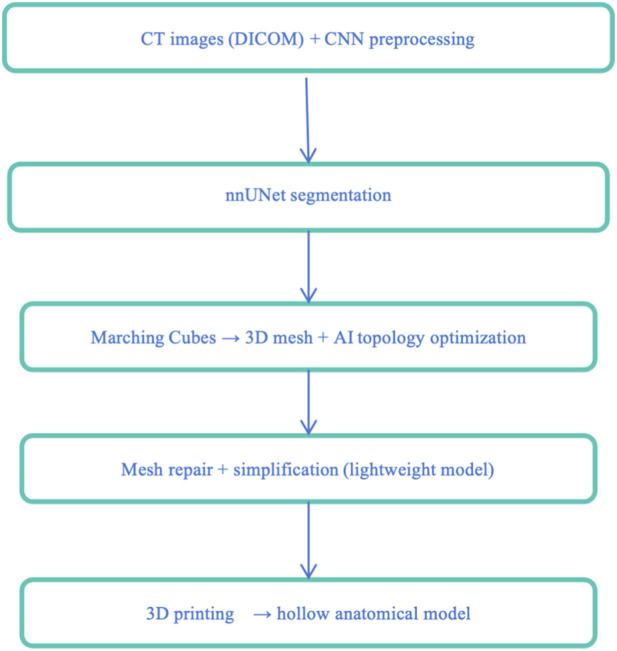
AI-assisted 3D printing pipeline for patient-specific upper gastrointestinal-biliary model.

**FIGURE 4:**
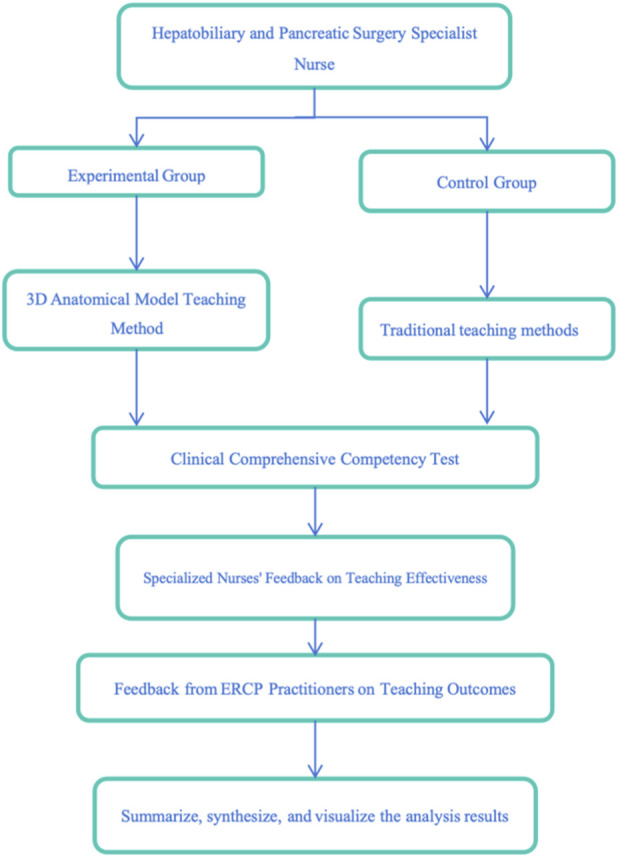
Flowchart of training teaching programs for both groups.

**FIGURE 5:**
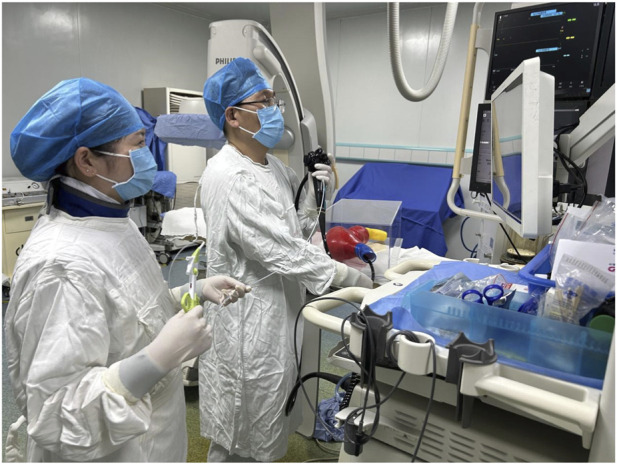
On-site photos of 3D-Printed model application in ERCP specialized nurse training.

1. With informed consent, thin-slice CT images (DICOM) were acquired from a patient with common bile duct stones, cholangitis, and an excessively acute gastroduodenal angle. CNN-based algorithms performed denoising, Z-score intensity standardization, and isotropic resampling to eliminate equipment differences and motion artifacts, ensuring data consistency. A pre-trained deep segmentation network (nnUNet) then performed pixel-level classification, automatically identifying and segmenting key anatomical structures (esophagus, stomach, duodenum, intrahepatic and extrahepatic bile ducts, gallbladder, and common bile duct) and outputting high-precision binary masks evaluated by Dice coefficient (Mowry et al., 2025). Surface rendering using Marching Cubes generated preliminary 3D meshes, followed by AI-assisted topology optimization to ensure continuity and anatomical rationality. The rough meshes underwent further post-processing: non-manifold structure repair eliminated staircase artifacts, and feature-preserving mesh simplification reduced polygon count while preserving anatomical details, producing lightweight, high-quality 3D models suitable for real-time interaction. Finally, a Stratasys Connex3350 printer fabricated hollow reconstructed upper gastrointestinal-biliary system models (Figures 1–3).
2. Four hepatobiliary surgeons from this center with over 10 years of clinical and teaching experience evaluated the models. Evaluation focused on morphological fidelity of key anatomical structures (gastric body morphology, duodenal morphology, duodenal papilla, and pancreaticobiliary junction), spatial relationship accuracy, and tissue texture realism. Experts unanimously agreed that the models demonstrated clear stratification and strong landmark features, possessing high academic and application value in complex anatomical teaching, preoperative simulation, and physician-patient communication.
3. Experimental group trainees received 4 weeks of clinical practical teaching centered on 3D-printed anatomical models, twice weekly for 2 hours per session. The teaching objective was to enable trainees to intuitively master duodenal papilla position and morphological characteristics, three-dimensional bile duct trajectories, and surrounding anatomical relationships through repeated observation, palpation, and simulated cannulation operations on anatomically abnormal upper gastrointestinal-biliary system models, while enhancing clinical collaboration abilities through team coordination simulation training. Control group trainees received traditional teaching methods over the same period and schedule, primarily through systematic instructor lectures with two-dimensional anatomical images in PowerPoint presentations and surgical video demonstrations. Their teaching objectives focused on enabling trainees to master and understand basic theoretical knowledge of normal and anatomically variant digestive-biliary systems and routine nursing coordination procedures through audiovisual memory. After the training period, unified assessment evaluations were conducted for both groups. Assessment tools included clinical comprehensive competency tests and teaching quality questionnaires. Clinical comprehensive competency tests encompassed theoretical written examinations (assessing anatomical knowledge, operational procedures, complication management, etc.) and practical assessments (completing specific ERCP coordination tasks in simulated environments, with a fixed ERCP team forming an examiner panel scoring trainees' operational standardization, proficiency, and teamwork according to standardized scoring rubrics). Teaching quality questionnaires employed Likert five-point scales to collect trainees' subjective evaluations of teaching methods regarding teaching model, teaching content, stimulating learning interest, enhance operational skills, and strengthening analytical and problem-solving abilities (Figures 4, 5). The questionnaire was adapted from a previously validated instrument for simulation-based nursing education, with modifications made to fit the ERCP training context (Fu et al., 2020) (see Supplementary Materials S1-3). Post-training, an ERCP examiner panel unaware of group assignments (single-blind) provided teaching effectiveness feedback for trainees, covering preoperative preparation, cooperation awareness, procedural competence, procedural accuracy, and teamwork awareness.

### 2.3 Statistical analysis

Statistical analyses were performed using R software (version 4.3.1). Continuous variables (theoretical and practical scores) are expressed as mean ± standard deviation (SD) and were compared between the experimental and control groups using Welch’s two-sample t-test, which does not assume equal variances. Cohen’s d and its 95% confidence interval (CI) were calculated for these outcomes using the t-based approximation.

For Likert-scale questionnaire outcomes (ordinal data), the Mann-Whitney U test was applied, and results are presented as median (interquartile range, IQR) together with U and Z statistics. Categorical data (e.g., gender, education level) were analyzed using the χ^2^ test. A two-sided *p* < 0.05 was considered statistically significant.

This study was initially designed as an exploratory investigation; therefore, no formal *a priori* sample size calculation was performed. After the final sample was expanded to 179 participants, a *post hoc* power analysis was conducted based on the observed effect size for the primary outcome (practical score, Cohen’s d = 1.21). With a two-sided significance level of α = 0.05, the achieved power (1-β) exceeded 99%, confirming that the sample size was adequate to detect the observed differences. For a moderate effect (d = 0.5), the power remained above 90%.

## 3 Results

Examination Scores: Pre-training and post-training theoretical knowledge and operational skills of nurses were scored out of 100 points. Theoretical knowledge was assessed through written examinations covering ERCP anatomical fundamentals, operational skill knowledge, and case-related operational knowledge.

### 3.1 Comparison of post-training theoretical and practical assessment scores between groups

Comparison of theoretical and practical assessment scores between experimental and control group nursing personnel is shown in Table 2. As shown in Table 2, the experimental group demonstrated significantly higher theoretical assessment scores (92.39 ± 3.41 vs. 90.31 ± 4.49, t = 3.523, *p* < 0.05) and practical assessment scores (93.30 ± 3.20 vs. 87.31 ± 5.28, t = 9.291, *p* < 0.05) compared to the control group.

**TABLE 2:** Comparison of post-training theoretical and practical assessment scores between groups.

Statistic	Theoretical score	Practical score
EG (n = 84)	92.39 ± 3.41	93.30 ± 3.20
CG (n = 95)	90.31 ± 4.49	87.31 ± 5.28
MD	2.09	5.99
95% CI of MD	(0.92, 3.26)	(4.72, 7.27)
Cohen’s d	0.528	1.391
95% CI of d	(0.229, 0.826)	(1.064, 1.719)
T	3.523	9.291
Df	173.2	157.6
P-value	<0.001*	<0.001*

EG: experimental group; CG: control group; MD: mean difference; CI: confidence interval. *: comparison between groups, *p* < 0.05.

### 3.2 Nurse feedback on teaching effectiveness

Post-training, experimental and control group nursing personnel evaluated training content across dimensions including Teaching Model, Teaching Content, Stimulating Learning Interest, enhancing operational skills, and strengthen analytical and problem-solving abilities. The questionnaires employed five-point Likert scales (from very dissatisfied to very satisfied, scored 1–5). Nurse feedback on teaching effectiveness is shown in Table 3. As shown in Table 3, in the teaching effectiveness feedback, the experimental group scored significantly higher than the control group in teaching model [median (IQR): 4 (4–4) vs. 3 (3–4); U = 5379, Z = 4.015, *p* < 0.001], stimulating learning interest [4 (3–4) vs. 3 (3–4); U = 5111.5, Z = 3.242, *p* = 0.0012] and enhancing operational skills [4 (3–4) vs. 3 (3–4); U = 4967.5, Z = 2.825, *p* = 0.0047]. No significant difference was observed in teaching content (*p* = 0.3513), Strengthening analytical and problem-solving abilities (*p* = 0.0629).

**TABLE 3:** Comparison of teaching effectiveness feedback between groups (Likert 1–5).

Statistic	Teaching model	Teaching content	Stimulating learning interest	Enhancing operational skills	Strengthening analytical and problem-solving abilities
EG (n = 84) median (IQR)	4 (4–4)	3 (3–3)	4 (3–4)	4 (3–4)	3 (3–4)
CG (n = 95) median (IQR)	3 (3–4)	3 (2–3)	3 (3–4)	3 (3–4)	3 (3–3)
U	5379	4312.5	5111.5	4967.5	4633.5
Z	4.015	0.932	3.242	2.825	1.860
P-value	<0.001*	0.3513	0.0012*	0.0047*	0.0629

Data are presented as median (interquartile range). U: Mann-Whitney U statistic; Z: standardized test statistic. *: Comparison between groups, *p* < 0.05.

### 3.3 ERCP physician feedback on teaching effectiveness

After the training period, unified assessments were conducted for both groups. To ensure objectivity and clinical relevance, three specialist physicians from this center with over 5 years of independent ERCP operation experience served as the evaluation expert panel for teaching effectiveness feedback. All evaluation experts were unaware of group assignments (single-blind):All participants were assigned unique study codes before the assessment. The expert panel was aware only of these codes and had no knowledge of the corresponding group allocation (experimental or control). Neither the training instructors nor the experts involved in the evaluation participated in the teaching intervention. The assessment was conducted in a simulated surgical environment, where each participant performed a standardized ERCP coordination task together with a member of the expert panel acting as the endoscopist. After the procedure, the expert panel members independently completed a self-developed questionnaire based on the participants' on-site performance. The expert panel then completed custom questionnaires to provide feedback on trainees' teaching effectiveness, using Likert five-point scales (from very dissatisfied to very satisfied, scored 1–5). Coordination satisfaction included five competencies: preoperative preparation, cooperation awareness, disposal ability, procedural accuracy, and teamwork awareness, totaling 50 points. As shown in Table 4, regarding ERCP physician feedback, the experimental group received significantly higher ratings than the control group in cooperation awareness [median (IQR): 4 (4–4) vs. 4 (3–4); U = 5230.5, Z = 3.585, *p* < 0.001], procedural accuracy [3 (3–4) vs. 3 (2–3); U = 5216, Z = 3.544, P < 0.001], and teamwork awareness [4 (4–4) vs. 4 (3–4); U = 5058, Z = 3.087, *p* = 0.002]. No significant differences were observed in preoperative preparation (U = 4480, Z = 1.416, *p* = 0.1567) or disposal ability (U = 4557.5, Z = 1.640, *p* = 0.1009).

**TABLE 4:** ERCP physician feedback on nursing teaching effectiveness (Likert 1–5).

Statistic	Preoperative preparation	Cooperation awareness	Disposal ability	Procedural accuracy	Teamwork awareness
EG (n = 84) median (IQR)	4 (4–4)	4 (4–4)	4 (4–4)	3 (3–4)	4 (4–4)
CG (n = 95) median (IQR)	4 (3–4)	4 (3–4)	4 (3–4)	3 (2–3)	4 (3–4)
U	4480	5230.5	4557.5	5216	5058
Z	1.416	3.585	1.640	3.544	3.087
P-value	0.1567	<0.001*	0.1009	<0.001*	0.002*

Data are presented as median (interquartile range). U: Mann-Whitney U statistic; Z: standardized test statistic. *: Comparison between groups, *p* < 0.05.

## 4 Discussion

Since its first report by McCune et al., in 1968, Endoscopic Retrograde Cholangiopancreatography (ERCP) has evolved from a purely diagnostic technique into an important minimally invasive modality for treating biliopancreatic diseases. With continuous innovation in endoscopic instruments and increasingly mature operative techniques (Rezapour et al., 2022), ERCP’s application scope has continuously expanded, now serving as the preferred treatment for common bile duct stones, benign and malignant biliary obstruction, chronic pancreatitis, and other conditions. Its advantage lies in accessing the biliopancreatic system through natural orifices, avoiding laparotomy, significantly reducing patient trauma, and shortening recovery time. However, during ERCP procedures, patients' individualized anatomical abnormalities pose severe challenges to operator technique and team coordination, with excessively acute gastroduodenal angles being particularly common and problematic. This anatomical feature disrupts the conventional gastric antrum-duodenal axis relationship, making endoscope advancement along natural curves difficult and readily forming non-functional large loops within the stomach, preventing precise force transmission to the distal tip. This causes unstable endoscope positioning in the duodenal descending portion, with papilla visualization significantly fluctuating with intestinal peristalsis and respiration, while severely distorting cannulation instrument axes, making alignment with common bile duct trajectories difficult and rendering conventional cannulation techniques ineffective. Operators must repeatedly attempt adjusting scope position, changing specialized pre-bent instruments, or using guidewire exploration, not only substantially prolonging operative time and increasing radiation exposure but also significantly elevating risks of mucosal injury, perforation, and post-procedure pancreatitis (Kılıç et al., 2025). Therefore, facing such anatomical variations, ERCP has transformed from standardized operations into complex technical challenges requiring highly individualized responses, demanding operators possess extensive adaptive experience and instrument control capabilities while testing assisting nurses' anticipation of and coordination with operators' non-standard procedural flows. Inadequacy at any stage may directly impact surgical success and patient safety. Intraoperative nurses must promptly comprehend operator movements, accurately deliver instruments, assist with papilla cannulation, manage patient conditions, and rapidly coordinate responses when complications such as bleeding or perforation occur. Previous studies have shown that physician-nurse coordination directly influences ERCP success rates and complication incidence (Shin et al., 2025). Nurses lacking systematic training and anatomical knowledge reserves not only struggle to effectively coordinate procedures but may also cause treatment delays or errors leading to prolonged operative times, increased radiation exposure, or even serious medical incidents (Ma et al., 2023).

Currently, domestic and international ERCP specialized nurse training predominantly employs combinations of theoretical lectures, on-site observation, and simulated operations (Tripathi et al., 2025). However, traditional training models have obvious limitations: on one hand, digestive system anatomical structures are complex, especially with numerous hepatic hilar bile duct and upper gastrointestinal variations and concealed duodenal papilla positions, making two-dimensional images inadequate for conveying true spatial relationships; on the other hand, cadaveric specimens are scarce, and animal models differ significantly from humans, leaving trainees without realistic, repeatable operative training conditions. Therefore, developing efficient, realistic, and ethically compliant training methods has become an urgent need for standardized ERCP specialist training.

In recent years, deep integration of artificial intelligence technology with medical imaging has brought revolutionary advances to three-dimensional anatomical modeling (Issa et al., 2023). Particularly in the digestive system field, AI-based CT image segmentation and reconstruction technology has significantly enhanced model generation efficiency and precision. The core of AI-assisted three-dimensional reconstruction lies in utilizing deep learning algorithms to automatically identify and segment target structures in CT images. The nnU-Net framework can adaptively configure network parameters and optimize training processes, achieving high-performance segmentation even with limited data samples. The specific workflow includes: first preprocessing raw CT data from anatomically abnormal patients, including grayscale standardization, denoising, and isotropic resampling; subsequently utilizing pre-trained models for pixel-level segmentation of upper gastrointestinal and biliary systems, distinguishing vessels, bile ducts, pancreatic ducts, and surrounding organs; finally generating three-dimensional mesh models suitable for 3D printing through surface rendering techniques. Digital models reconstructed using AI can be manufactured into high-fidelity physical models through multi-material 3D printing technology (Teles de Campos et al., 2025).Flexible photosensitive resins can simulate digestive tract soft texture, while transparent materials display internal duct trajectories and interrelationships. Such models possess the following educational value: Providing tactile feedback: trainees can directly touch and manipulate models to understand common digestive tract variations, sphincter region texture, papilla hardness, and resistance during instrument cannulation—experiences irreplaceable by image-based learning (Shibuki et al., 2025). Supporting repeated operative training: models possess good durability and cleanability, suitable for multiple cannulation and stent placement practice sessions. Enhancing spatial cognition: through multi-angle observation and detachable design, trainees can deeply understand complex three-dimensional relationships such as biliopancreatic duct confluence patterns and papilla opening positions.

In the field of simulation-based ERCP nursing training, a domestic research team has confirmed that a virtual reality endoscopy simulator combined with micro-teaching methods can improve nurses’ procedural success rates and reduce completion times (Gangwani et al., 2024). In a more recent study, a silicone ERCP training model developed using 3D printing technology—for example, a portable simulator created by an Asian team—has been shown to significantly enhance physicians’ skills in both basic and advanced procedures, including duodenoscope insertion, common bile duct cannulation, and plastic stent placement (Force et al., 2024). To the best of our knowledge, however, no study has systematically applied an AI-assisted, patient-specific 3D-printed model to ERCP nurse training targeting the specific anatomical variation of an excessively acute gastroduodenal angle, nor has any study validated the effectiveness of this educational approach through a large-sample randomized controlled trial.

Integrating AI-assisted 3D-printed models into ERCP nurse training can form more systematic, efficient teaching models. This enhances learning initiative and immersion: trainees can first identify papillary structures on printed models, then perform cannulation operations under instructor guidance. This approach significantly improves learning interest and participation. It strengthens theory-practice integration: in ERCP training, trainees must complete preoperative planning, instrument selection, and complication contingency planning based on models, thereby transforming abstract knowledge into clinical competence. Instructors can provide targeted guidance based on specific issues in trainee operations, avoiding learning risks in real patient operations. Although many large domestic and international endoscopy centers widely employ endoscopic simulators for physician-nurse training, and these simulators have played positive roles in familiarizing operational procedures and basic instrument use, their anatomical structures are typically based on standardized “textbook” designs, struggling to reproduce the wide range of anatomical variations encountered in clinical practice. The 3D-printed models employed in this study offer advantages rooted in “authenticity.” Through AI reconstruction of imaging data from anatomically abnormal patients, their high-fidelity, individualized anatomical reproduction enables trainees to encounter and adapt to various complex situations potentially encountered in clinical practice during training, thereby bridging the cognitive gap between generic models and real patients. This not only enhances trainee immersion and engagement but directly cultivates nurses' coordination abilities for specific clinical anatomical contexts—capabilities unmatched by fixed generic simulators. Therefore, this study’s 3D printing approach represents not a simple replacement of existing simulation training but an important upgrade in training precision and clinical relevance.

Study results show that the training group employing this model achieved significantly higher theoretical assessment scores and practical assessment scores compared to the control group. Simultaneously, training outcomes showed significantly higher satisfaction in Teaching Model, Stimulating learning interest and Enhance operational skills compared to the control group. Additionally, experimental group nurses demonstrated significantly higher ERCP physician feedback scores for Cooperation Awareness, Procedural Accuracy, and Teamwork Awareness satisfaction compared to the control group. These results indicate that the AI-3D printing-based individualized model training approach effectively promotes dual transformation and integration of explicit knowledge (theory) and tacit knowledge (operational skills). Training effectiveness is verified not only at the subjective perception level but also through objective quantitative assessments, confirming this model’s advantages in enhancing ERCP specialized nurses' comprehensive job competency. It represents an effective training method capable of transforming abstract anatomical knowledge into clinical practical abilities. This model innovates traditional teaching methods at the pedagogical level, promoting more active teaching interactions; at the learning psychology level, its intuitiveness and realism significantly enhance trainees' proactive exploration willingness; at the core skills level, it directly translates into higher operational ability satisfaction. Collectively, these findings demonstrate that this technology is not merely a novel teaching tool but a systematic educational intervention capable of simultaneously optimizing “pedagogy, learning motivation, and skill transformation.” Simultaneously, this training model effectively enhances nurses' clinical situational awareness and team integration capabilities, with value extending from individual skill levels to surgical team collaboration effectiveness. Physician evaluation improvements indicate that experimental group nurses, through individualized model training, not only mastered operational techniques but deeply understood operator intentions and procedural workflows, thereby achieving more precise instrument delivery, more anticipatory coordination, and more seamless teamwork in real surgeries, ultimately translating into directly perceptible coordination quality improvements for surgical physicians. This confirms this training model’s important role in cultivating “operator-centered” team surgical thinking.

Despite this model’s promising application prospects, several challenges remain: High cost and technical barriers: AI modeling requires high-performance computing equipment, while 3D printing involves material and equipment costs, with substantial initial investment. Simultaneously, mastering modeling and printing technology requires interdisciplinary knowledge, placing greater demands on faculty (Zhang et al., 2022). Model precision and realism still have improvement potential: current printing materials can simulate tissue texture but cannot perfectly reproduce dynamic physiological responses such as peristalsis and bleeding. AI segmentation precision requires further enhancement. Current evaluations of 3D-printed model teaching effectiveness rely primarily on operational assessments and questionnaires, lacking unified quantitative indicators to measure long-term teaching benefits and clinical translation value. Additionally, all participants were recruited from a single tertiary hospital, and the training environment and resources had inherent limitations. Although the study spanned several years (2020–2025), the training protocol, assessment tools, and the 3D-printed model remained unchanged throughout. Baseline characteristics did not differ between early and late enrollees (data not shown). Nevertheless, potential unmeasured temporal changes in institutional training practices cannot be completely excluded.Therefore, the generalizability of the study findings requires further validation through multi-center research. Although the sample size of 179 was sufficient to detect a moderate effect size (power >90%), the relatively homogeneous training setting may not fully represent the diversity of clinical nursing training practices across hospitals of different levels or in different geographic regions.Moreover, the teaching effectiveness questionnaire consisted of single-item dimensions, which precluded the calculation of internal consistency (Cronbach’s α). The practical-skills assessment was performed by a single examiner; therefore, inter-rater reliability was not evaluated. Future studies should consider using multi-item scales and independent raters to improve the psychometric robustness of the assessments.

This study not only validates individualized models' value in routine ERCP training but its methods and concepts possess broad applicability. For example, for other common anatomical variations affecting ERCP operations (such as giant duodenal diverticula, ampullary tumors, or pancreaticobiliary junction abnormalities), the “real case-AI reconstruction” pathway employed in this study can generate targeted training models. Particularly for patients who have undergone Billroth II gastrectomy or other upper gastrointestinal reconstructions, ERCP operative pathways are completely altered with extreme difficulty, posing severe challenges for endoscopists. By constructing anatomical models of such high-difficulty cases, junior operators can intuitively understand spatial relationships and operational challenges of variant anatomy in risk-free environments, systematically practicing specialized techniques such as retrograde cannulation. Simultaneously, assisting nurses can familiarize themselves with non-standard surgical layouts, instrument delivery sequences, and potential emergencies through models, thereby achieving precise, efficient coordination with operators in real surgeries. Therefore, this study provides a feasible technical solution and important practical insights for establishing a tiered, individualized ERCP training system covering common variations to extremely complex cases.

## 5 Conclusion

In this single-center exploratory study, AI-assisted 3D modeling of upper gastrointestinal-biliary systems with gastroduodenal angle variations showed potential advantages in ERCP specialized nurse training, including improved anatomical cognition, practical skill acquisition, and translation of knowledge into practice compared to traditional methods. These preliminary findings suggest that such individualized simulation models may serve as a useful adjunctive educational tool for nursing education. However, given the limited sample size (n = 179), single-center design, and absence of long-term outcome data, the results should be interpreted as hypothesis-generating rather than definitive. Further multi-center randomized controlled trials with larger cohorts and extended follow-up are necessary to validate the generalizability and durability of these observed benefits. At present, this approach represents an encouraging step toward more personalized simulation-based training, but its role in routine education and clinical practice requires additional evidence.

## Data Availability

The raw data supporting the conclusions of this article will be made available by the authors, without undue reservation.
