# Supplementary material for: Application and validation of AI-assisted 3D-Printed gastroduodenal anatomical variation models in specialized nursing training
Source: Front Bioeng Biotechnol. 2026 Jun 19;14:1769764. doi: 10.3389/fbioe.2026.1769764 (PMC13327972; doi:10.3389/fbioe.2026.1769764)
Supplement: Supplementary file 1 [file DataSheet2.pdf]

ERCP Specialized Nurse Operation Assessment Scoring Rubric

Trainee Code: __________ Score: __________

Assessment Item	Points	Scoring Criteria	Score
I. Preoperative Preparation (20 points)			
1. Environment and equipment check	3	Check function of endoscopic system, light source, high-frequency generator, X - ray machine, suction unit, monitor, oxygen supply, etc. (1 point); confirm availability of emergency medications and equipment (epinephrine, atropine, defibrillator, etc.) (1 point); inspect duodenoscope for leaks and cleaning/disinfection status (1 point).	
2. Instrument and supply preparation	5	Prepare according to surgical needs: sphincterotome, guidewires (hydrophilic / zebra), contrast catheter, retrieval basket/balloon, dilation balloon/bougie, nasobiliary tube, stents (plastic/metal), pancreatic stent, hemostatic clip, injection needle, lithotripter, etc. (2 points); check integrity and expiry dates (1 point); arrange in order of use (1 point); prepare contrast agent, saline, iced epinephrine - saline, lubricant, etc. (1 point).	
3. Patient assessment and verification	4	Verify patient identity, surgical site, informed consent (1 point); assess vital signs, SpO_2, IV access (1 point); confirm fasting time, presence of dentures/metal accessories (1 point); obtain	

		history of past illnesses, allergies, anticoagulant use and discontinuation status (1 point).	
4. Patient positioning and comfort	3	Assist patient into left lateral prone position (left arm behind back, right arm naturally placed), head turned to one side, mouth corner lower than pharynx (1 point); place soft cushions to protect bony prominences (1 point); adjust bite block and secure firmly (1 point).	
5. Intraoperative medication preparation	2	Prepare sedatives/anesthetics, antispasmodics (hyoscine butylbromide / glucagon), antibiotics, NSAIDs (indomethacin suppository), etc. (1 point); verify drug name, dose, and expiry (1 point).	
6. Personal preparation	3	Wear standard attire: cap, mask, goggles/face shield (1 point); perform hand hygiene, put on sterile gown and sterile gloves (1 point); wear lead apron, lead collar, and personal dosimeter (1 point).	
II. Intraoperative Cooperation (50 points)			
7. Cannulation assistance	10	Assist with scope advancement and angle control as needed (1 point); deliver sphincterotome / contrast catheter after papilla is exposed (1 point); connect contrast syringe, purge air, and hand to physician (1 point); gently advance guidewire, feel resistance, do not force (2 points); after guidewire passes papilla, assist with bile aspiration and low - pressure contrast injection (2 points); switch instruments as instructed (precut, double - guidewire, pancreatic stent, etc.) (2 points); record cannulation time and number of attempts (1 point).	

8. Guidewire/sphincterotome handling	8	Understand characteristics of different guidewires (straight - tip, angled, hydrophilic, zebra) and select as needed (1 point); lubricate guidewire tip and insert through instrument channel, maintain coaxial alignment (2 points); connect sphincterotome to high - frequency cord, ensure cutting wire is retracted and orientation correct before foot pedal activation (2 points); during cutting, observe wire tension, direction and depth, remind physician promptly (2 points); after cutting, assist with irrigation and observe for bleeding (1 point).	
9. Contrast assistance	4	Use low - concentration contrast (1 point); inject slowly with moderate pressure, avoid overfilling (1 point); observe filling sequence and morphology of bile/pancreatic ducts (1 point); take radiographs/record images in a timely manner (1 point).	
10. Stone extraction assistance	8	Select appropriate retrieval basket/balloon according to stone size (1 point); pass the device over the guidewire; after the physician crosses the stone, assist in opening the basket or inflating the balloon (2 points); withdraw slowly, instruct patient to hold breath (1 point); collect stone for analysis after extraction (1 point); assist with lithotripsy (mechanical/laser/electrohydraulic) if needed (2 points); repeat extraction until no residual stones on cholangiogram (1 point).	
11. Stent/nasobiliary tube placement	6	Measure stricture length and location, select appropriate stent (1 point); advance stent delivery system over guidewire, confirm	

		position under X - ray (1 point); during deployment, nurse stabilizes guidewire while physician slowly withdraws the outer sheath (1 point); after deployment, aspirate to confirm bile flow (1 point); for nasobiliary tube, assist with oral - to - nasal exchange using traction wire or guidewire (1 point); secure nasobiliary tube to cheek and connect drainage bag (1 point).	
12. Instrument exchange and transfer	6	Verbally repeat instrument name and size before transfer (1 point); maintain sterility during transfer, tip toward operator (1 point); ensure guidewire remains in place during exchange to avoid withdrawal (2 points); retrieve used instruments promptly to prevent contamination (1 point); anticipate next instrument during complex procedures to shorten time (1 point).	
13. Radiation protection	3	Use foot pedal appropriately, avoid unnecessary fluoroscopy (1 point); during fluoroscopy, personnel stay as far as possible from X - ray tube (1 point); use pulsed mode to shorten exposure time (1 point).	
14. Patient monitoring and communication	5	Continuously monitor vital signs, SpO_2 , ECG (1 point); observe patient ‘s color, consciousness, cough or hiccup (1 point); promptly clear oral and nasal secretions to prevent aspiration (1 point); reassure patient during procedure, instruct cooperation (deep breathing, relaxation) (1 point); if abnormality detected (hypoxia, hypotension, arrhythmia), immediately report and assist in management (1	

		point).	
III. Post - operative Management (12 points)			
15. Scope withdrawal and instrument handling	3	Assist in scope withdrawal, organize guidewires and instruments (1 point); sort instruments (reusable: initial rinse, send for sterilization; disposable: place in sharps container) (1 point); bedside pre - cleaning of endoscope (aspirate cleaning solution and enzymatic solution, send to reprocessing unit) (1 point).	
16. Patient transfer and handover	4	After confirming stable vital signs, assist transfer to recovery room or ward (1 point); hand over to receiving nurse with information on patient details, procedure, drainage patency, and any complications (2 points); provide post - operative instructions (fasting, bed rest, observe for abdominal pain/bleeding/fever, etc.) (1 point).	
17. Nursing documentation	3	Accurately record procedure details, instruments used, medications, complications, drainage status, etc. (2 points); complete billing and consumable records (1 point).	
18. Drainage tube care	2	Ensure nasobiliary tube / pancreatic stent / nasopancreatic tube is properly secured and clearly labeled (1 point); keep drainage bag below the insertion site, maintain patency (1 point).	
IV. Aseptic Technique and Infection Control (8 points)			
19. Aseptic technique	4	Strictly follow aseptic principles throughout the procedure (2 points); if contamination occurs, immediately change instruments or gloves (2 points).	

20. Hand hygiene	2	Perform hand hygiene before and after each patient contact and before/after aseptic procedures (2 points).	
21. Sharp and waste disposal	2	Place sharps directly into sharps container; dispose of waste in designated categories (2 points).	
V. Communication and Teamwork (5 points)			
22. Communication with physician	2	Clearly and accurately repeat physician ‘s instructions (1 point); proactively anticipate physician’ s next move and prepare accordingly (1 point).	
23. Communication with patient	2	Explain procedure in simple language to reduce anxiety (1 point); inform patient about expected sensations (bloating, nausea) and coping methods (1 point).	
24. Documentation and reporting	1	Verbally report key steps (successful cannulation, stone extraction, stent placement, etc.) and record them (1 point).	
VI. Emergency Response (5 points)			
25. Hypoxia	1	Immediately increase oxygen flow, perform jaw thrust, assist with mask ventilation or notify anesthesiologist as needed (1 point).	
26. Bleeding	2	Assist with local irrigation, prepare hemostatic clips, sclerosing agent, epinephrine solution (1 point); monitor blood pressure and heart rate, establish IV access, prepare blood transfusion (1 point).	
27. Perforation	1	Immediately stop the procedure, assist with clip or covered stent placement (1 point).	
28. Contrast allergy	1	Administer anti - allergic medication as ordered, assist in resuscitation (1 point).	
Total	100	Total Score	

ERCP 专科护士操作考核评分细则

考生编码：__________　得分：__________

考核项目	分值	评分标准	得分
一、术前准备 (20 分)			
1. 环境与设备检查	3	检查内镜主机、光源、高频电发生器、X 线机、吸引器、监护仪、氧气等设备功能（1分）；确认急救药品及器材（肾上腺素、阿托品、除颤仪等）在位（1 分）；检查十二指肠镜测漏、清洗消毒状态（1 分）。	
2. 器械物品准备	5	根据手术需求准备：切开刀、导丝（亲水/斑马）、造影导管、取石网篮/气囊、扩张球囊/探条、鼻胆管、支架（塑料/金属）、胰管支架、止血夹、注射针、碎石器等（2 分）；检查器械完整性及有效期（1 分）；按使用顺序摆放（1 分）；准备造影剂、生理盐水、去甲肾上腺素冰盐水、润滑剂等（1 分）。	
3. 患者评估与核对	4	核对患者身份、手术部位、知情同意书（1 分）；评估生命体征、血氧饱和度、静脉通路（1 分）；确认禁食禁饮时间、有无义齿/金属饰品（1 分）；了解既往史、过敏史、抗凝药物使用史及停药情况（1 分）。	
4. 患者体位与舒适度	3	协助患者取左侧俯卧位（左臂置背后，右臂自然），头部偏向一侧，口角低于咽部（1分）；垫软枕保护骨隆突处（1 分）；调节牙垫位置固定牢固（1 分）。	
5. 术中用药准备	2	备镇静/麻醉药、解痉药（丁溴东莨菪碱/胰高血糖素）、抗生素、非甾体抗炎药（吲哚美辛栓）等（1 分）；核对名称、剂量、有效期（1 分）。	
6. 个人准备	3	着装规范，戴帽、口罩、护目镜/面屏（1 分）；手卫生，穿无菌手术衣，戴无菌手套（1分）；佩戴铅衣、铅围脖、个人剂量计（1 分）。	
二、术中配合 (50 分)			
7. 插管配合	10	医师进镜时协助扶镜、控制角度钮（1 分）；乳头暴露后递送切开刀/造影导管（1 分）；连接造影剂注射器排气后交医师 (1 分)；导丝插入轻推、感受阻力，不强行推进 (2 分)；导丝越过乳头后协助回抽胆汁、低压注射造影剂（2 分）；根据指令切换器械（预切开、双导丝、胰管支架等）(2 分)；记录插管时间及次数（1 分）。	
8. 导丝/切开刀使用配合	8	熟悉导丝 (直头/弯头/亲水/斑马) 特性按需选择 (1 分)；导丝前端润滑后经器械腔送入，保持同轴（2 分）；切开刀连接高频电导线，踩脚踏前确认刀丝收拢、方向正确（2 分）；切开过程中观察刀丝张力、切割方向及深度，及时提醒（2 分）；切开后协助冲洗创面，观察出血（1 分）。	
9. 造影配合	4	使用低浓度造影剂（1 分）；缓慢推注，压力适中，避免过度充盈（1 分）；观察胆/胰管显影顺序及形态（1 分）；及时拍照/录像记录（1 分）。	
10. 取石配合	8	根据结石大小选网篮/气囊（1 分）；取石器经导丝送入，医师越过结石后护士协助张开网篮/充盈气囊（2 分）；缓慢回拉，嘱患者暂停呼吸（1 分）；结石拖出后收集送检（1分）；必要时配合碎石（机械/激光/液电）(2 分)；重复取石至造影无残留（1 分）。	
11. 支架/鼻胆管放置配合	6	测量狭窄长度及位置，选合适支架 (1 分)；沿导丝推送支架推送器，X 线下确认位置（1分）；释放支架时护士固定导丝，医师匀速后退外鞘（1 分）；释放后回抽见胆汁流出，确认通畅（1 分）；鼻胆管放置时协助经口鼻交换（牵引丝/导丝引导）(1 分)；固定鼻胆管于面颊部，连接引流袋（1 分）。	
12. 器械交换与传递	6	传递器械前口头复述名称及规格（1 分）；传递时保持无菌，尖端朝向术者（1 分）；交换器械时确保导丝固定在位，避免退出（2 分）；器械用后及时收回，避免污染（1 分）；	

		复杂操作时预判下一器械，缩短时间（1 分）。	
13. 放射防护	3	合理使用脚踏，避免不必要的透视（1 分）；透视时人员尽量远离球管（1 分）；使用脉冲模式，缩短曝光时间（1 分）。	
14. 患者监护与沟通	5	持续监测生命体征、血氧、心电图（1 分）；观察面色、意识、有无咳嗽或呃逆（1 分）；及时清理口鼻腔分泌物，防误吸（1 分）；术中安抚患者，指导配合（深呼吸、放松）（1 分）；发现异常（低氧、低血压、心律失常）立即报告并协助处理（1 分）。	
三、术后处理 (12 分)			
15. 撤镜与器械处理	3	协助撤镜，整理导丝、器械（1 分）；器械分类收集（可复用初洗送消毒；一次性入锐器盒）（1 分）；内镜床旁预处理（吸引清洗液、酶液，送洗消室）（1 分）。	
16. 患者转运与交接	4	评估生命体征稳定后协助转运至复苏室/病房（1 分）；与接收护士交接患者信息、手术过程、引流通畅情况、有无并发症（2 分）；交代术后注意事项（禁食、卧床、观察腹痛/出血/发热等）（1 分）。	
17. 护理记录	3	准确记录手术过程、器械使用、用药、并发症、引流情况等（2 分）；填写费用、耗材登记（1 分）。	
18. 引流管护理	2	鼻胆管/胰管支架/鼻胰管妥善固定，标识清晰（1 分）；引流袋低于引流口，保持通畅（1 分）。	
四、无菌操作与感染控制（8 分）			
19. 无菌技术	4	操作过程严格遵守无菌原则（2 分）；污染后立即更换器械或手套（2 分）。	
20. 手卫生	2	每次接触患者前后、无菌操作前后执行手卫生（2 分）。	
21. 锐器与废弃物处理	2	锐器直接放入锐器盒，废弃物分类放置（2 分）。	
五、沟通与团队协作（5 分）			
22. 与医师沟通	2	清晰、准确复述医师指令（1 分）；主动预判医师下一步操作并提前准备（1 分）。	
23. 与患者沟通	2	用通俗语言解释操作过程，减轻紧张（1 分）；告知术中感觉（腹胀、恶心等）的正常性及应对方法（1 分）。	
24. 记录与报告	1	关键步骤（插管成功、取石完成、支架放置等）口头报告并记录在案（1 分）。	
六、应急处理能力（5 分）			
25. 低氧血症	1	立即增加氧流量，托下颌，必要时协助面罩加压或通知麻醉医师（1 分）。	
26. 出血	2	配合局部冲洗，备止血夹、硬化剂、肾上腺素液（1 分）；监测血压、心率，开放静脉通路，备血（1 分）。	
27. 穿孔	1	立即停止操作，协助放置夹子或覆膜支架（1 分）。	
28. 造影剂过敏	1	遵医嘱给抗过敏药物，协助抢救（1 分）。	
总分	100	合计得分	
