# Supplementary material for: Application and validation of AI-assisted 3D-Printed gastroduodenal anatomical variation models in specialized nursing training
Source: Front Bioeng Biotechnol. 2026 Jun 19;14:1769764. doi: 10.3389/fbioe.2026.1769764 (PMC13327972; doi:10.3389/fbioe.2026.1769764)
Supplement: Supplementary file 2 [file DataSheet3.pdf]

ERCP Physician Feedback Form on Nurse Teaching Effectiveness

Trainee Code: __________

Instructions: Please rate the nurse ' s intraoperative cooperation performance during ERCP by ticking (✓) the corresponding score for each dimension.

Scoring criteria: 1 = Very dissatisfied, 2 = Dissatisfied, 3 = Neutral, 4 = Satisfied, 5 = Very satisfied.

Dimension	1	2	3	4	5
1. Preoperative preparation					
2. Cooperation awareness					
3. Disposal ability					
4. Procedural accuracy					
5. Teamwork awareness					
6. Overall satisfaction					
Open - ended suggestions (optional):					

ERCP 诊疗医师对护士教学效果反馈情况表

考生编码：__________

填表说明：请您根据该护士在 ERCP 术中配合的真实表现，对以下各维度进行评价，在相应分数下打“√”。
评分标准：1=非常不满意，2=不满意，3=一般，4=满意，5=非常满意。

评价维度	1 分	2 分	3 分	4 分	5 分
1. 术前准备					
2. 配合意识					
3. 处置能力					
4. 操作准确性					
5. 团队配合意识					

6. 总体满意度
开放式建议（选填）：
