# Supplementary material for: Application and validation of AI-assisted 3D-Printed gastroduodenal anatomical variation models in specialized nursing training
Source: Front Bioeng Biotechnol. 2026 Jun 19;14:1769764. doi: 10.3389/fbioe.2026.1769764 (PMC13327972; doi:10.3389/fbioe.2026.1769764)
Supplement: Supplementary file 3 [file DataSheet1.pdf]

ERCP Specialized Nurse Training Teaching Quality Questionnaire

Dear Trainee,

To continuously improve the quality of ERCP specialized nurse training, please rate the following dimensions based on your actual experience during this training. This questionnaire is anonymous and the results will be used only for teaching improvement. Thank you for your cooperation!

Instructions: Please tick (✓) the corresponding score for each item. Scoring criteria: 1 = Very dissatisfied, 2 = Dissatisfied, 3 = Neutral, 4 = Satisfied, 5 = Very satisfied.

**Dimension**	**1**	**2**	**3**	**4**	**5**
1. Teaching model					
2. Teaching content					
3. Stimulating learning interest					
4. Enhancing operational skills					
5. Strengthening analytical and problem-solving abilities					
6. Overall satisfaction					

Open-ended questions (optional):

What do you think was the greatest strength of this training?

What suggestions do you have for future ERCP nurse training?

Thank you again for your participation!

ERCP 专科护士培训教学质量问卷调查表

尊敬的学员：

您好！为了不断提高 ERCP 专科护士培训质量，请您根据本次培训的真实感受，对以下各维度进行评价。本问卷采用匿名方式，结果仅用于教学改进，感谢您的配合!

填表说明：请在每项后面相应的分数下打“√”。评分标准：1=非常不满意，2=不满意，3=一般，4=满意，5=非常满意。

评价维度	1 分	2 分	3 分	4 分	5 分
1. 带教模式					
2. 教学内容					
3. 激发学习兴趣					
4. 提高操作能力					
5. 增强分析和解决问题能力					
6. 总体满意度					

开放式问题（选填）：

您认为本次培训最大的亮点是什么?

您对今后 ERCP 护士培训有哪些建议?

再次感谢您的参与!
